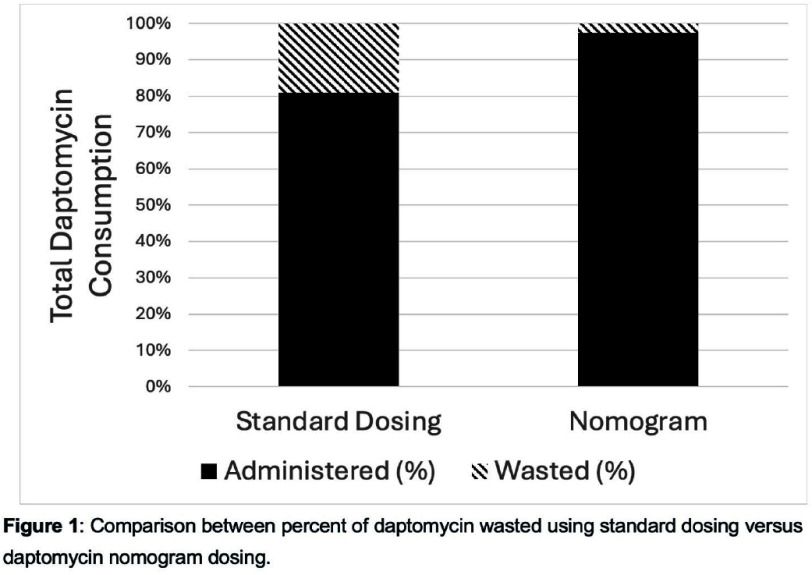# Optimizing Daptomycin Dosing: Environmental Benefit and Cost Savings

**DOI:** 10.1017/ash.2025.193

**Published:** 2025-09-24

**Authors:** Pamela Lee, Marina Nguyen, Michelle LeBrun, Gary Fong, Loren Miller

**Affiliations:** 1 Harbor UCLA Medical Center; 2 Rancho Los Amigos; 3 Harbor-UCLA Medical Center

## Abstract

**Background:** The pharmaceutical industry is estimated to have a larger environmental footprint than the automotive industry. Discarded and unused doses of pharmaceuticals generate financial waste and pollution, and exacerbate antibiotic shortages. The antibiotic daptomycin is dispensed in standard-sized single-use vials and dosed based on patient weight. Residual daptomycin in the vial after dose preparation must be disposed of and cannot be used for another patient. We hypothesized that daptomycin dosing nomogram use would reduce daptomycin waste, environmental impact, and financial costs. **Methods:** We performed a retrospective chart review quantifying daptomycin waste, defined as disposed of unused daptomycin, at Harbor-UCLA Medical Center, a 400-bed Level 1 Trauma Center, from 1/1/2023 to 12/31/2023. We then adjusted dosing using a daptomycin dosing nomogram. We modeled the difference in daptomycin waste (mg of daptomycin disposed of unused), pharmaceutical waste (weight of excess daptomycin vials required due to wasted antibiotic), and cost between the two dosing strategies. Our model assumed a daptomycin vial weight of 16.8g and cost of $30 per 500mg daptomycin vial. We conservatively estimated pharmaceutical waste as waste only from daptomycin vials, ignoring all other supplies and materials necessary to prepare daptomycin. **Results:** During the 1 year time period at our Medical Center, 138,882mg daptomycin was wasted. This level of daptomycin waste equates 4671g excess pharmaceutical waste and $8332 spent on unused, discarded daptomycin. In our model, we found that nomogram implementation would have reduced mean monthly daptomycin waste from 11,002mg to 1387mg (p<0.001). This reduction would have decreased the proportion of daptomycin wasted from a mean of 19% to 3% of all consumed daptomycin (Figure 1). Nomogram use would also have saved $7333 and averted 4111g of pharmaceutical waste in 2023. **Conclusion:** A daptomycin dosing nomogram would have prevented 122,322mg of daptomycin from being wasted and saved over $7000 at a 400 bed Medical Center over one year. Given the 4111 g of pharmaceutical waste is a conservative estimate, and ignores waste from other supplies/materials as well as upstream waste and emissions from daptomycin manufacturing, the overall generated environmental impact prevented by nomogram use is likely significantly higher. Our findings demonstrate that intentionally designed dosing strategies aimed at reducing drug waste can save hospital costs and reduce the environmental footprint of clinical care. When implemented at large health systems these strategies are likely to result in substantial cost savings and reduction in the negative environmental impact associated with pharmaceuticals.

**Figure:**